# Assessing Survival and Grading the Severity of Complications in Octogenarians Undergoing Pulmonary Lobectomy

**DOI:** 10.1155/2017/6294895

**Published:** 2017-02-08

**Authors:** Andrew Feczko, Elizabeth McKeown, Jennifer L. Wilson, Brian E. Louie, Ralph W. Aye, Jed A. Gorden, Eric Vallières, Alexander S. Farivar

**Affiliations:** ^1^Division of Thoracic and Foregut Surgery, Swedish Medical Center and Cancer Institute, 1101 Madison Street, Suite 900, Seattle, WA 98104, USA; ^2^Surgical Specialists of Charlotte, 2001 Vail Ave., Suite 320, Charlotte, NC 28207, USA; ^3^Department of Surgery, Chest Disease Center, Beth Israel Deaconess Medical Center, 185 Pilgrim Road, Suite 201, Boston, MA 02215, USA

## Abstract

*Introduction.* Octogenarians are at increased risk for complications after lung resection. With alternatives such as radiation, understanding the risks of surgery and associated survival are valuable. Data grading the severity of complications and long-term survival in this population is lacking. We reviewed our experience with lobectomy in octogenarians, grading complications using a validated thoracic morbidity and mortality schema.* Methods*. We retrospectively reviewed consecutive patients aged ≥80 undergoing lobectomy between 2004 and 2012. Demographics, clinical/pathologic stage, complications, recurrence, and mortality were collected. Complications were graded by the Seely thoracic morbidity and mortality model.* Results*. 45 patients (mean age 82.2 years) were analyzed. The majority of patients (28/45, 62%) were clinical stage IA/IB. 62% (28/45) of patients experienced a complication. Only 15.6% (7/45) were considered significantly morbid (≥ grade IIIB) per the Seely model. Perioperative mortality was 2% and half of patients were living at a follow-up of 53 months. Overall five-year survival was 52%.* Conclusions*. In carefully selected octogenarians, lobectomy carries a 15.6% rate of significantly morbid complications with encouraging overall survival. These data provide the basis for a more complete discussion with patients regarding lobectomy for lung cancer.

## 1. Introduction

Due to the high incidence of lung cancer in octogenarians, surgeons are increasingly confronted with the clinical challenge of how best to treat these patients with resectable lung cancer. Fourteen percent of patients diagnosed with lung cancer in the United States from 1988–2003 were ≥80 years old [1] with the peak incidence occurring between ages 75–79 [2]. With the general population living longer and the baby boomer generation reaching this age bracket in the next decade, it is imperative that we better understand complications and survival in this growing age group.

Making optimal treatment recommendations can be challenging for treating physicians as octogenarians in general have more comorbidities and a worse performance status than the younger population. While the gold standard surgical therapy for early stage lung cancer remains anatomic resection [3], physicians may be hesitant to refer these patients for surgical evaluation and other treatment options including stereotactic body radiation therapy (SBRT) and wedge resection may be suggested [4]. Currently, long-term survival data for SBRT are limited and show a wide range of survival ranging from a 3-year survival of 42% for stage I [5] to 83% for stage IA [6] (see Table 4). In addition, the surgical literature regarding octogenarians with lung cancer is diverse and often includes patients with a wide variety of clinical stages and resections (i.e., wedge, lobectomy, and pneumonectomy). As a result, broad 5-year survival rates ranging from 18.2% to 69.6% [7–17] have been reported which are likely strongly related to variable inclusion data and extent of resection (see Table 3).

Prior publications have documented that octogenarians experience higher complication rates after lung resection which ranges from 8.4 to 68.8% [7–14, 16–19]. Data regarding the severity, morbidity, or impact of those complications has yet to be elucidated using a standardized validated system, leaving physicians with an incomplete understanding of assessing short-term surgical risks versus long-term benefit. Furthermore, surgeons lack the data that allow them to characterize morbidity and complications beyond “major or minor” when counseling their patients. In order to reconcile this, the Seely morbidity and mortality schema was designed for application to thoracic surgical patients [20]. It provides a standardized and validated framework for physicians to describe complications based on the level of intervention required [20].

Defining complication severity is important in the over-eighty population to allow for accurate patient counseling regarding surgical outcomes and to guide surgical treatment algorithms. We sought to gain further understanding of the inherent morbidity assumed by an octogenarian deemed appropriate for lobectomy and to characterize complications based on a validated thoracic surgery schema. Furthermore, we assessed long-term survival to contextualize more completely short-term risks versus long-term survival.

## 2. Methods

We performed a single center retrospective review of consecutive patients aged eighty or greater who underwent lobectomy approached by thoracotomy, video assisted thoracoscopic surgery (VATS), or robotic assisted methods between 2004 and 2012. All patients were staged according to the American Joint Committee on Cancer (AJCC) 7th edition guidelines [21]. The Institutional Review Board (IRB) approved the study and data was entered into a secure database. Individual patient consent was waived due to the retrospective nature of the study.

Key data elements included age, gender, body mass index (BMI), comorbidities, percent of predicted forced expiratory volume expired in 1 second (FEV1), percent of predicted diffusion capacity of carbon dioxide (DLCO), operative approach, conversion of minimally invasive operative approach to thoracotomy, mean operative time, mean estimated blood loss, intraoperative mortality, complications, intensive care unit (ICU) stay, clinical and pathologic stage, procedure performed, hospital length of stay (LOS), posthospital patient disposition to home or skilled nursing facility (SNF), follow-up duration, disease recurrence, patient status (living or deceased), and cause of death if applicable.

Patients were staged as follows. Cross sectional imaging was used to determine tumor size and clinical T stage (cT). Clinical node negative status (cN0) was determined if the patient had a standardized uptake value (SUV) <1.5 on positron emission tomography (PET), a biopsy of a PET positive node that was ultimately pathologically benign, or biopsy of lymph node(s) > 1 cm in largest diameter that was pathologically benign.

Each attending surgeon determined patient operability based on the patient's preoperative imaging, medical comorbidities, activity tolerance, pulmonary function testing, and nutrition status. Specifically, this included patients with an ECOG score of 0 or 1, a FEV1 and DLCO > 40% predicted after lobectomy, and no anginal symptoms. Patients with a significant cardiac history or questionable cardiac reserve were referred to their cardiologist or newly evaluated for clearance to tolerate a surgery and general anesthetic. Each patient performed an in-office stair walk where heart rate and oxygen saturation were recorded while walking at least two flights of stairs. Patients who became significantly dyspneic or had an oxygen saturation less than 92% while walking two flights were considered inoperable. No comorbidity precluded consideration for an operation; however, care was taken to optimize these conditions (i.e., blood glucose and pulmonary function) prior to consideration of surgery. This assessment determined the ability of a patient to tolerate general anesthetic and a lobectomy through any modality. Once operability was determined, the decision to proceed with a thoracotomy or minimally invasive (VATS or robotic) procedure was at the surgeon's discretion.

We employed the Seely classification system, which characterizes complication severity based on the need for general anesthesia separating minor from major complications. Grade I complications include any complication not requiring intervention; grade II complications include any complication requiring pharmacologic intervention; grade IIIA complications include any intervention not requiring general anesthesia. Major complications were defined as grade IIIB (any complication requiring general anesthesia), grade IV (any complication requiring ICU admission, single organ failure, or reintubation), and grade V (complications leading to death) (see Table 2).

Clinical follow-up was calculated from the date of discharge to the last clinic visit. In cases where a patient was lost to follow-up, status was verified by using the social security death registry and/or by contacting their primary care physician. Survival data was analyzed by the Kaplan-Meier method. A univariate analysis was performed to identify factors that influence overall survival including medical comorbidities, clinical and pathologic stages, tumor size, and complications (all and severe ≥IIIB). Statistical analyses were completed using SPSS 18.

## 3. Results

A total of 45 (male = 23) patients underwent lobectomy. No patients received neoadjuvant therapy prior to resection. The most common comorbidity was hypertension (56%) (see Table 1). All patients were high functioning with an ECOG of 0 (39/45, 86.7%) and 1 (6/45, 13.3%). The majority of patients (42/45, 93.3%) underwent preoperative cardiac evaluation by a cardiologist. Nearly all patients were clinically staged with a diagnostic chest CT and PET. Thirty-nine patients (86.7%) underwent preoperative cervical mediastinoscopy. Clinical staging information was available for 41/45 patients. Of these, 20 were stage IA (51%), 8 stage IB (21%), 5 stage IIA (11%), 1 stage IIB (2.2%), and 5 stage IIIA (11%). Two patients underwent lobectomies for metastasectomy, and staging information was unavailable for the remaining four patients. All patients undergoing lobectomy for lung cancer were clinically node negative and without evidence of metastasis (cN0 and cM0) prior to resection.

Thoracotomy was performed most commonly (27/45, 60.0%) followed by VATS (12/45, 26.7%) and robotic assisted (3/45, 6.7%). In two cases (2/15, 13.3%), a minimally invasive approach was converted to thoracotomy. Mean operative time was 228 minutes (range 106–381 minutes) and mean estimated blood loss was 50 mL. There was no intraoperative mortality. Disposition data was available for 32/45 patients and 27/32 (84.4%) were admitted to the ICU postoperatively. The average ICU LOS was 3 days (range 1–13).

The pathology for most patients was non-small-cell lung cancer (NSCLC) (39/45, 86.7%) and the majority of these were adenocarcinoma (26/39, 66.7%). Two patients had metastatic disease from an extrathoracic source (colorectal and uterine stromal cell carcinoma) and 2 patients had a spindle cell neoplasm (one bronchioloalveolar carcinoma and one neuroendocrine tumor). In keeping with the early clinical stage of this population, the most common pathologic stage in the NSCLC patients was IB (13/39, 33.3%). Ten patients (10/39, 25.6%) were IA; 3 patients were IIA (3/39, 7.7%); 3 patients were IIB (3/39, 7.7%); 7 patients were IIIA (7/39, 18%); 1 patient was IIIB (1/39, 2.6%), and 2 patients were Stage IV (2/39, 5.1%) on final pathology.

Minor complications (grades I–IIIA) occurred in 46.7% (21/45) of patients. New onset atrial fibrillation requiring medical therapy was the most common complication (22.2%, 10/45). Ten patients (22.2%) had a prolonged air leak or fluid collection that necessitated chest tube for > 5 days. Nine of these air leaks resolved while the patient was in hospital, but 1 patient was discharged home with a chest tube due to a persistent leak. Four patients (8.9%) were discharged from the hospital with supplemental oxygen therapy per nasal cannula.

Severe postoperative complications (≥IIIB) occurred in 15.6% (7/45) of patients. Three patients (6.7%) developed pneumonia requiring ICU care. Two patients (4.4%) required return to the operating room for control of postoperative hemorrhage within twenty-four hours of the initial operation. Additional severe complications included myocardial infarction (2/45, 4.4%) and one inpatient sustained an ankle fracture due to a fall, which subsequently required operative fixation (see Table 2). Furthermore, 1 patient (2.2%) with steroid-dependent COPD died in the perioperative period on postoperative day 13 due to sepsis induced multiple organ system failure.

Over half of the patients had a postoperative complication of some sort (28/45, 62%). Additional complications not captured in the Seely classification system included unexpected transfers to the ICU for cardiac monitoring due to atrial fibrillation which occurred in 3/45 (6.7%). Thirty-six of the 45 patients had disposition data available. Of these patients, 88.9% (32/36) were able to be discharged home and 11.1% (4/36) were discharged to a skilled nursing facility.

During the mean follow-up period of 53 months (range 1 week to 84 months), 13.3% (6/45) had disease recurrence at a mean of 25 months from their operation (4 months–84 months) and 51.1% (23/45) died. Causes of death included lung cancer recurrence in 43.5% (10/23), multiorgan system failure in the perioperative period, stroke, amiodarone toxicity, and sepsis (1 each). Nine patients died of unknown causes. Mean 5-year overall survival calculated by the Kaplan-Meier method was 52%. Medical comorbidities, clinical stage, tumor size, any complication (grades I–V), and severe complications (≥IIB) did not significantly influence survival on univariate analysis.

## 4. Discussion

While anatomic lung resection remains the standard of care for the treatment of NSCLC [3], octogenarians are less likely to be offered a lobectomy than younger patients. A Surveillance, Epidemiology and End Results (SEER) database review of 45,912 of patients aged ≥80 demonstrated that octogenarians were twice as likely to receive no local tumor specific therapy (radiation or surgery) when compared to younger patients despite a comparable rate of early stage cancers in both groups [1]. This was even significant when comparing octogenarians to patients aged 70–79 (47% offered no radiation or surgery versus 28%) [1]. Another study demonstrated the same trend and showed that as age increases, patients undergo anatomic resection less often than the general population and are offered radiation therapy more often than surgery [22] despite the fact that radiation therapy or nonanatomic lung resection remains second line therapy for patients with operable NSCLC [3].

Even with improvement in radiation methods from external beam radiation therapy, stereotactic body radiation (SBRT), or stereotactic ablative radiation therapy (SABR) [23], resection offers the best chance for local control [24]. In studies including octogenarians who are surgically or medically inoperable, treatment decisions are a bit easier for healthcare providers. This study sought to understand and clarify the risks and outcomes of the gold standard therapy (resection) within this group.

There has been a broad attempt across the surgical literature to classify complications by severity in order to standardize complication reporting. The National Cancer Institute Common Terminology Criteria for Adverse Events (CTCAE) is a system which grades adverse events across multiple diverse areas of medical care on a 1–5 scale [25]. The validated Clavien-Dindo complication classification scheme was created for application to general surgery patients and is graded based upon the level of intervention required to treat the complication, a more practical system than simply separating events into major and minor categories [19]. Seely et al. published a validated extension of the Clavien-Dindo system for application to the thoracic surgery patient population [20]. Like the CTCAE, complications are graded from I to V based on the level of intervention required to treat the complication, with minor complications considered grades I-II and major complications that result in significant morbidity listed as grades III–V [20]. We used Seely grade in order to provide us with a specific understanding of the practical morbidity faced by octogenarian population when undergoing lung resection.

Lower % predicted FEV1 [19], thoracotomy as approach [9, 19], resection greater than wedge [19, 26], and mediastinal lymph node dissection [11, 16] are associated with higher complication rates in octogenarians undergoing lung resection. In addition, Port et al. demonstrated a statistically significant shorter length of stay and less ICU admission rates with VATS over thoracotomy in octogenarians undergoing lobectomy [9]. While we did not seek to find differences in operative approach in this study, we did not observe a difference in complication rates between thoracotomy, VATS, and robotic, likely due to the study size. Minimally invasive approaches to lung resection have been shown to be associated with fewer complications by many authors [9, 19] and should be considered for octogenarians if appropriate.

Long-term survival after lobectomy in the elderly population is an important consideration. The calculated 5-year overall survival of 52% in our series was comparable to previous reported 5-year overall survival of 18.2–69.6% [7–17]. Furthermore, for stage IA patients the calculated 5-year overall survival rate was excellent at 78% compared to 69.6% reported previously [9]. Univariate analysis in our study failed to reveal predictors of better survival aside from lower pathologic stage. In this study, there was no statistically significant survival benefit dependent on operative approach.

We have observed that many of our patients are concerned with the possible need for a nursing facility (SNF) stay postoperatively. For patients in whom disposition data were available (36/45), 32 (88.9%) were able to be discharged home with 4 (11.1%) requiring discharge to a SNF. These rates are similar to other studies which report that 6% [19] and 16.5% [9] of octogenarians require SNF after lung resection.

Limitations to this retrospective review include patient selection bias, lack of a control group, small size, and variability of operative approach. The classification of complications as minor or major depending on level of intervention required does not reflect patient quality of life and the perceived invasiveness of the intervention. Additionally, these results may not be reproduced at other centers with different patient selection criteria and experience. Our follow-up was also limited at 1.7 years.

In today's society, where an 80 year old male can expect to live an additional 8 years (an 80-year-old female can live another 9.3 years) [27], carefully selected medically fit patients over 80 should be offered anatomic resection for early stage, operable NSCLC as per the NCCN guideline recommendations [3]. We found that employing an intervention-based, validated complication classification scheme is helpful when discussing expected surgical outcomes within this population (octogenarians) and across studies.

We have also shown that appropriately chosen octogenarians can undergo lobectomy with encouraging overall survival. These data should help physicians and surgeons understand the short-term risks and long-term survival associated with lobectomy, provide a framework for future comparison to SBRT or wedge resection, and allow surgeons to counsel older patients more thoroughly.

In conclusion, while 62% of octogenarians undergoing lobectomy had a complication, severe complications using the Seely morbidity and mortality complication schema occurred in only 15.6% of our study group. In addition, octogenarians deemed operable by a thoracic surgeon, regardless of stage or surgical approach, can achieve a reasonable 5-year survival of greater than 50%. Using this data as a foundation, providers can accurately counsel similar patients regarding their surgical complication risk and expected survival.

## Figures and Tables

**Table 1:** Patient demographics.

Mean age (range)	82.2 (80–89)
Mean BMI (range)	25 (17–43)
Mean FEV1% predicted (range)	86 (43–123)
Mean DLCO% predicted (range)	71 (42–110)
Tobacco history: *n* (%)	39 (87%)
Mean pack years	30
Comorbidities: *n* (%)	
HTN	25 (56%)
CAD	14 (31%)
COPD	12 (27%)
Prior CT surgery	12 (27%)
Atrial fibrillation	10 (22%)
Diabetes	3 (7%)
Renal disease	1 (2%)
Steroid dependent	1 (2%)

BMI: body mass index, FEV1: forced expiratory volume at one second, DLCO: diffusing capacity for lung for carbon monoxide, HTN: hypertension, CAD: coronary artery disease, CT: cardiothoracic, and COPD: chronic obstructive pulmonary disease.

**Table 2:** Complications categorized by the Seely thoracic morbidity and mortality classification system.

Grade	Patients *n*/45 (%)^*∗*^	Definition of complication	Complication description (*n*/48, %)^*∗∗*^
I	2 (4.4%)	Complication that does not require any intervention	Asymptomatic vocal cord paralysis (1, 2.1%), urinary retention (1, 2.1%), ileus (2, 4.2%)

II	10 (22.2%)	Pharmacologic therapy or minor intervention required	Atrial fibrillation (14, 29.2%, with 10 being new onset; 10/48, 20.8%^*∗∗∗*^), esophagitis (2, 4.2%), new home O_2_ (4, 8.3%), serious electrolyte disturbance (1, 2.1%), acute kidney injury (1, 2.1%), chyle leak (1, 2.1%)

IIIA	9 (20%)	Interventions not requiring general anesthesia	Home with chest tube (1, 2.1%) stroke (1, 2.1%), bleeding not requiring transfusion (1, 2.1%), development of a PTX requiring drainage (1, 2.1%), chest tube duration > 5 days (10, 20.8%)

IIIB	3 (6.7%)	Interventions requiring general anesthesia	Return to OR: postoperative hemorrhage (2, 4.2%), fracture fixation after fall (1, 2.1%)

IV	3 (6.7%)	Complication requiring ICU support, reintubation, single or multisystem organ failure	MI (2, 4.2%), pneumonia (4, 8.3%), respiratory failure (1, 2.1%)

V	1 (2.2%)	Any complication leading to death	Multiorgan failure (1, 2.1%)

^*∗*^Several patients had more than 1 complication and are listed in the category corresponding to the complication with the highest Seely grade.

^*∗∗*^Complications are listed individually in this column and many include multiple complications in the same patient(s).

^*∗∗∗*^The four patients with preexisting atrial fibrillation were not included in the complication rate.

O_2_: oxygen, OR: operating room, and MI: myocardial infarction.

**Table 3:** Lung resection in octogenarians: comparative data.

Author	Years of enrollment	*N*	Approach (T, V, R)	Resection(s) included (W, S, L, B, P)	Age	Clinical stages included	Pathologic stage	Complication rate (% overall), severe	Predictors of increased complications (*p* values)	30-day perioperative mortaiity (%)	5-year survival	Predictors of poor survival (*p* value)
Port et al. [9]	1998–2009	121	V = 40 T = 81	L	≥80 (median 82)	—	I 65.3% II 25.0% III 10.0%	53.7%, 28.9% severe requiring significant intervention	Thoracotomy (63%) versus VATS (35%) *p* = 0.004	1.7%	56.6% By stage: I = 69.6% II = 35.6% III = 18.2%	Pathologic stage Ib or greater (*p* = 0.050)

Hanagiri et al. [18]	1992–1995	18	T	S, L, W	≥80 (mean, 82.1)	IA 6 IB 6 IIB 1 IIIA 5	IA 4 IB 5 IIB 4 IIIA 1 IIIB 4	50%	—	0%	—	—

Aoki et al. [7]	1981–1998	35	—	W or S 10 L 25	≥80 (mean 81.2)	IA 14 IB 10 IIA 0 IIB 5 IIIA 5 IIIB 1	—	60%	—	0%	39.8%	—

Pagni et al. [8]	1980–1995	54	T	W 3 S 3 L 43 B 2 P 1 chest wall 1 sleeve 1	≥80	I II IIIA	—	42%, 11%	—	3.7%	43%	>stage I (*p* value not given)

Clavien et al. [19]	2000–2009	191	V 77% T	W 77 S 13 L 96 P 3	≥80 (median 82)	—	I 56% II 10% III 9% IV 3%	46%	Resection greater than wedge (*p* = 0.0064), thoracotomy as approach (*p* = 0.034), % predicted FEV1 for ea 10% decrement (*p* = 0.01)	3.6%	3-year stage I (109 patients) 56%	—

Umezu et al. [10]	2001–2008	44			≥80 (mean 82)			65.9%		2.3%	54.5%	

Okada et al. [17]		44			≥80 (mean 81.8)			20%		0	44.9%	

Okami et al. [11]	1999	367	—	W 80 S 42 L 245	80–90 (mean 82)	I	I 300 II 44 III 23	8.4%	Comorbidity and mediastinal lymph node dissection	1.4%	56.1%	Advanced pathologic stage and comorbidity

Chida et al. [16]	1981–2006	48	—	S 3 L 45 P 2	≥80 (mean 81.7)	I 36 II 3 III 9	I 30 II 8 III 10	68.8%	Mediastinal LN dissection (*p* = 0.004)	—	35%	Med LN dissection (*p* = 0.022)

Suemitsu et al. [15]	1981–2006	146	—	W 38 (26%) S 17 (11.6%) L 79 (54.1%) B 7 (4.8%) P 1 (0.7%) Explorations 4 (2.7%)	≥80 (mean 82.6)	I 109 (74.7%) II 14 (9.6%) III 22 (15.1%) IV 1 (0.7%)	I 94 (64.3%) II 16 (11.0%) III 31 (21.2%) IV 5 (3.5%)	—	—	—	46.8%	—

Mun and Kohno [14]	1999–2006	55	V	W 10 (18.2%) S 7 (12.7%) L 37 (67.3%) B 1 (1.8%)	80–89 (mean 82.7)	IA 32 IB 23	I 44 II 6 III 5	25.6%	—	3.6%	65.9%	—

Ikeda et al. [13]	1981–2002	73	V and T	L 45 (62%) B 2 (2.7%) S 6 (8.2%) W 20 (27%)	80–89 (mean 83)	I 60 II 10 IIIA 3	I 55 (74.3%) II 8 (11%) IIIA 5 (6.8%) IIIB 4 (5.5%) IV 1 (1.4%)	37%	—	4.1%	47%	Pathologic stage III compared to II (*p* = 0.02)

Matsuoka et al. [12]	1997–2004	40	85% Muscle sparing PLT/VATS, 15% standard PLT	W 30% S 30% L 40%	80–89	—	IA 52.5% iB 35.5% IIA 0 IIB 7.5% IIIA 5%	20%	—	0%	56.9%	—

Current Study	2004–2012	45	T 59% V 26% R 26%	L	≥80 (mean 82)	IA 51% IB 21% IIA 11% IIB 2.2% IIIA 11%	IA 26% IB 33% IIA 8% IIB 8% IIIA 18% IIIB 3% IV 5%	62%, 17%	—	2%	52%	—

**Table 4:** SBRT: comparative Data.

Author	Years of enrollment	Total Gy (fractions)	*N*	Age (median)	Clinical stages included	Follow-up (median months)	Complications (NCI-CTC grade)	3-year primary tumor control rate	3-year local and regional control	3-year rate of disseminated failure	DFS	Overall Survival	Predictors of poor survival (*p* value)
Nagata et al. [6]	1998–2004	48 (4)	45	51–87	Stage I: T1N0M0 T2N0M0	30	4% (2)	—	—	—	3 year IA 72% IB 71%	3 year IA 83% IB 72%	—

Dales et al. [28]	2006–2009	54 (3)	55	48–89 (72)	Stage I: T1N0M0 T2N0M0	34.4	12.7% (3) 3.6% (4)	97.6%	87.2%	22.1%	3 year 48.3%	3 year 55.8% (median 48.1 mo)	—

Matsuo et al. [24]	1999–2005	48 (4)	66	67–86 (76.5)	Stage I: T1aN0M0 T1bN0M0 T2aN0M0	35.9	—	—	—	—	5 year 33.8%	5 year 44.6% (median 35.9 mo)	—

Fakiris et al. [5]	—	60–66 (3)	70		T1N0M0 T2N0M0	50.2	8.6% (3) 1.4% (4) 7.1% (5)	88.1%	—	—	—	3 year 42.7% (median 32.4 mo)	—

Timmerman et al. [29]	2004–2007	48 (4)	65	50–91 (median 89)	T1N0M0	45.4	9.2% (3)	—	—	—	—	3 year 76%	—

Nagata et al. [30]	1999–2007	—	875	75–97	Stage I: T1N0M0 T2N0M0	—	—	—	—	—	—	—	—

Palma et al. [31]	1995–2004	100–141 Gy	87	74	Stage I: T1N0M0 T2N0M0	55	1.1%	—	—	—	—	5 yr 72% (S1a), 62% (S1b)	—

Current Study	2004–2012	—	45	≥80 (mean 82)	Stages I–IIIA	53	62% overall, severe 17% Seely grade ≥IIIB	—	—	—	—	5 year 52%	—
